# The Health of the English Soldier

**Published:** 1858-04

**Authors:** 


### SANITARY REVIEW.

APRIL 1858.

### THE HEALTH OF THE ENGLISH SOLDIER.

A WEIGHTY blue book lies before us on the subject of the health of the English soldier. The book has created lasting interest, and has given rise, even in the brief time that has elapsed since its issue, to several useful and necessary reforms. In the first pages of this REVIEW we drew attention to many of the facts recorded in the official document in hand; and in a special memorial to Lord Panmure, to which the names of several of the Commissioners who have taken part in the present inquiry were appended, we published what may be considered as the pith and the original stuff of all that has now been brought forward in relation to the sanitary requirements of our army.

But the report has taken the public by surprise. Its revelations, which are really but utterances in detail of similar revelations made years long since, and insisted on in general terms by at least a hundred writers past and present, falls now on the public ear with a startling sound. Why it has thus acted it is not difficult to understand. The people have been making themselves acquainted with military men and military affairs. They have felt in these days the value of their armed protectors. They have been making themselves acquainted with the wants of the soldier. They have not forgotten the Crimea, and its eventful horrors. Therefore the time is as ripe for this report, as the report is ripe for time. In addition, the book bears the stamp and seal of authority. Nunky tells what nunky pays. It is a great disadvantage, however, that the report, as a work to be bought, is too expensive to come into the hands of more than a favoured few. We have found, indeed, in conversation with numerous folk respecting this report, that they know about it only from hearsay or secondhand reading. To meet the wants of the community we shall, therefore, before offering a word of comment, give an abridgement of the report itself, supplying every important fact and suggestion of a sanitary kind offered by the commissioners, and following their mode of arranging the information (which for once, in a blue book, is exceedingly happy) as closely as is compatible with our space and intention.

"*Rates of Mortality in the Army.* No principle in vital statistics is better established than that the rates of mortality differ widely among different ranks, classes, and occupations, and that such difference is permanent, when arising from permanent causes.

"It seemed to us, therefore, important to ascertain the rates of mortality incidental to the soldier's life, as compared with those of trades in which men of the same class and the same age are usually engaged, and the conditions of which bear the greatest resemblance to his own occupation.

"We must, however, premise that the army rate of mortality cannot be correctly calculated from the deaths per 1,000 of the troops during the period they are serving, because every year a certain per-centage of the force is invalided and pensioned, on account of disease contracted in the service; and of men so pensioned, a large per-centage die during the first year. The health of the army, as given in the returns, is therefore more favourable in appearance than in reality, on account of the constant discharge from its ranks of men in an advanced stage of disease, as we shall presently show.

"But if the rates of mortality of the soldier, even with this qualification, exceed those of the classes with which he is compared, then the causes of that excess should be investigated, and the means of removing them, or, if removal be impossible, of mitigating their intensity, be sought.

"The soldier is recruited generally at 19 years of age. He is drawn from two classes, the agricultural labourer and the working class in towns. He is carefully inspected by a surgeon, who rejects every man having any bodily infirmity, or bearing signs of future disease. Although the soldier's is, therefore, a picked life, we propose before contrasting the rates of the mortality to which he is subject with those of some special classes and occupations, to compare them with those of the male population generally at corresponding ages throughout England and Wales, as stated by the Registrar-General."

There is then supplied a Table which shows—

"That whereas the deaths in the general English male population at the army ages during the fifteen years from 1839 to 1853, calculated at the annual average of 9·2 per 1,000, would have been 16,211, the deaths in the British army at home and abroad were 58,139, being an excess of deaths among soldiers of no less than 41,928.

"It may, however, be fairly objected to this comparison, that the civilians in England are living in their own country, and in a temperate and healthy climate; whereas a large portion of the army is serving in every part of the globe, and is exposed to every vicissitude both of temperature and climate.

"The following tables are free from that objection, and show the comparative mortality of the army at home, and of the male civil population of England and Wales, between the same ages as the soldier, irrespective of the occupations in which they are engaged, and of the healthiest and unhealthiest portions of it, as stated by the Registrar-General.

*Rates of Mortality per* 1000 *per Annum.*

| | |
|---|---|
| Effective men of all ages of the army at home: | |
| Total | 17·5* |
| Household Cavalry | 11·0 |
| Dragoon Guards and Dragoons | 13·3 |
| Foot Guards | 20·4 |
| Infantry of the Line | 18·7 |
| Population of England and Wales, army ages: | |
| Town and country population | 9·2 |
| Country alone | 7·7 |
| One of the unhealthiest towns, army ages: | |
| Manchester | 12·4 |

*Rates of Mortality per* 1000 *Men of the Army at home, and of the English Civil Male Population, at corresponding quinquennial periods, as stated by the Registrar-General.*

| | | |
|---|---|---|
| Ages, 20 to 25, | Civilians | 8·4 |
| | Soldiers | 17·0 |
| 25 to 30, | Civilians | 9·2 |
| | Soldiers | 18·3 |
| 30 to 35, | Civilians | 10·2 |
| | Soldiers | 18·4 |
| 35 to 40, | Civilians | 11·6 |
| | Soldiers | 19·3 |

"From this it appears, that if the army at home were as healthy as the population from which it is drawn, soldiers would die at one-half the rate at which they die now.

"But, as we have already observed, the soldier's is a picked life. All men offering to enlist, who bear signs of physical weakness or of tendency to disease, are rejected, and, even after acceptance, can be discharged on the representation of the regimental surgeon at any period within three years from their admission; and all these rejected lives are thrown back on the civil population.

"The apparent health of the army is maintained by the continued influx of fresh lives in the place of those which are weeded out by the process of invaliding, by which means a large number of men, whose physical powers are exhausted, are thrown back on the civil population, while their removal lowers the rates of mortality of the army, though their deaths are owing to the military service which first undermined their health.

"Thus, if all corps were exposed to the same risks, and the invaliding in each were conducted on the same principle and with the same degree of stringency, the rate of mortality would be low in each arm of the service in proportion as the invaliding is high,

* The cause of the difference between these numbers and those given in the Army Statistical Report is, that the above are corrected for the numbers in the service at different ages.

and *vice versâ;* and the rate of mortality among the invalids themselves would be highest where the amount of invaliding was the lowest, and *vice versâ.* The following table, however, shows that, owing to different disturbing causes, the results produced are at variance with the rule above stated.

| | Annual Ratio discharged by Invaliding per 1000 serving. | | | | |
|---|---|---|---|---|---|
| | Under 7 Years Service. | 7 to 14 Years Service. | 14 to 21 in Infantry, and 24 in Cavalry. | Total und. 21 or 24 Years Service. | Above 21 or 24 Years Service. |
| Household Cavalry | 6·2 | 13·6 | 35·1 | 15·2 | 531·3 |
| Cavalry of the Line serving at home | 14·7 | 23·5 | 36·7 | 20·9 | 739·0 |
| Foot Guards | 14·3 | 16·1 | 20·0 | 15·9 | 331·4 |
| Infantry of the Line serving at home | 15·9 | 21·1 | 55·6 | 20·8 | 987·0 |

"To the State, the loss of the men by invaliding is the same as the loss by death. In either case the expense of obtaining and training a substitute must be incurred, and in either case the death of the man, whether he be a soldier in the receipt of pay, or an invalid in the receipt of pension, is equally attributable to military service.

"It is obvious, therefore, that the rates of mortality, taken alone, represent a part only of the loss annually caused in the ranks of the army by disease.

"We have been enabled to institute some comparisons between the rates of mortality of the soldier and those of civilians of different classes and occupations, the materials of which, so far as civil life is concerned, have been placed before us by Mr. Neison.

"It must be borne in mind that a great part of the soldier's duty is performed in the open air; that he receives, as compared with the agricultural classes, an ample supply of food, is well clothed, and is housed at a considerable expense.

"In all these respects, looking at his material position alone, his condition at first sight appears to resemble that of the agricultural labourer, though the latter has disadvantages from which the soldier is secured. If the agricultural labourer, not being a member of a friendly society, falls sick, he forfeits his wages, which are his only means of subsistence. His medical treatment is not lavish, nor the attendance in all cases immediate. If he be a member of a friendly society he has better attendance, and his sick pay puts him beyond the reach of privation.

"To the soldier the government stands in the place of the friendly society. If he be ill, however slight the malady, he at once goes to hospital, and has all the treatment and all the nourishment which his case may require.

"Materially speaking, therefore, the life of the soldier at home (and in the following comparisons we are speaking only of the troops in the United Kingdom) and that of the agricultural labourer who is a member of a friendly society, would seem to be open to a fair comparison.

"The tables contained in the able paper by Mr. Neison show, however, that within corresponding ages the

| | | |
|---|---|---|
| Mortality of the Household Cavalry is | = 1·8 | times as great as the mortality of agricultural labourers being members of friendly societies;* |
| " Dragoons, etc. - | = 2·2 | |
| " Line - - | = 2·9 | |
| " Guards - - | = 3·3 | |

which is only 6·056 deaths per annum in 1,000 as against 11·1 per 1,000 in the Household Cavalry, 13·5 in the Dragoons, 17·9 in the Infantry of the Line, and 20·4 in the Foot Guards.

"But it may be objected, that though the soldiers' are originally picked lives, and though the government secures to them the same advantages as those provided for their members by friendly societies, yet the moral element in the character of that labourer whose providence induces him to lay by from his wages against the future, gives him an advantage in a comparison with the army taken as a body, without reference to the habits of the individual soldier.

"The man who is provident is probably likewise industrious and sober, and his standard of health higher than that of men who have not the same qualities in a like degree. As regards this condition of providence, which is a moral condition, acting on a man's physical state, those soldiers only who are depositors in the Regimental Savings' Bank should be compared with the agricultural members of friendly societies. Unfortunately, however, the statistics of the army do not afford the materials for instituting such a comparison, though we think it most desirable that in future they should be so arranged.

"The results of comparisons between the rates of mortality of soldiers and the different classes and occupations, are placed in juxtaposition in the following table :—

Deaths per 1,000 per Annum at Ages between 20 and 40.

| Agricultural labourers. Members of Friendly Societies. | Labourers. Rural districts. Whether members of F.S. or not. | Out-door trades in towns requiring great or little exercise. Not F.S. | Trades in towns only partially out-door. Not F.S. | Printers. Not F.S. | Police. Not F.S. | Miners. Not F.S. | Household Cavalry. | Dragoon Guards and Dragoons. | Infantry of the Line. | Foot Guards. | Artillery. |
|---|---|---|---|---|---|---|---|---|---|---|---|
| 6·055 | 8·002 | 8·538 | 8·449 | 9·090 | 8·922 | 10·314 | 11·1 | 13·5 | 17·8 | 20·4 | — |

* In these comparisons with various civil occupations or societies, it must be remembered that the persons engaged in them frequently withdraw, thus reducing the rates of mortality of the occupation which they leave; but against this must be set the discharge and invaliding of soldiers; the withdrawal, however, in the former case being at the will of the employed, in the latter at the will of the military authorities.

"The occupation which comes nearest to the army as regards its rate of mortality, is that of clerks. The close application to business, the sedentary attitude, the want of exercise and of fresh air, render their employment one of the most unhealthy of all extensive occupations.

"It seems almost incredible that it should be necessary to have recourse to the most unhealthy occupations in order to institute any comparison in which the rates of mortality shall approximate to those prevailing among your Majesty's troops, for at present the army stands almost at the head of unhealthy occupations in the United Kingdom.

"It is true, that though these comparisons between the army and various occupations in civil life have been confined to the troops quartered at home, yet, a large proportion of the latter having served abroad, sometimes in unhealthy climates, such as India and the West Indies, many have had the seeds of disease implanted in them during their tropical service. That many of these stations are extremely prejudicial to health is clearly shown.

"But the Guards, it must be observed, who have the highest rates of mortality of all the troops serving in the United Kingdom, do no Indian nor colonial duty, and the majority of the cavalry serves almost as exclusively at home.

"The following table shows the deaths per 1,000, on different stations in the navy and army, from 1830 to 1836:—

| | Navy. | Army. |
|---|---|---|
| On the home station - - - - | 8·8 | 13·7 |
| Mediterranean, including, in navy, the peninsular command - | 9·2 | 18· |
| Naval forces in the East India command and troops in Ceylon | 15·1 | 46·2 |

"But a certain proportion of this superiority on the part of the navy is accounted for by the larger amount of invaliding in that service than in the army, as will be seen from the following table:—

| | No. of men invalided per 1000. | |
|---|---|---|
| | Navy. | Army. |
| Home station - - - - | 38·2 | 25·3 |
| Mediterranean - - - - | 25·7 | 9·5 |
| East India command and Ceylon - - | 33·6 | 3·6 |

"The following table, for a later period, shows the mortality *from disease alone* in the navy, in the seven years, 1837-43:—

| | Per 1000 Annually. | |
|---|---|---|
| Home - - - | 6·8 | |
| South America - - | 6·7 | |
| Various - - - | 8· | |
| Packet Service - - | 8·6 | |
| North Coast, Spain - | 8·5 | Four years average only. |
| Mediterranean - - | 10·7 | |
| Cape - - - | 11· | |
| West Indies - - | 19·2 | Unhealthy stations. |
| East Indies - - | 34·2 | Unhealthy stations. |
| West Coast, Africa - - | 57·0 | Unhealthy stations. |
| Annual average per 1000 - | 14·9 | Mean force 33,000. |

"From this it appears that, though on the most unhealthy station, the mortality reaches 57 per 1,000, among the seamen at home, who generally are young men entering the service, it is less than that of the general population at the same ages; but it must be remembered that the disadvantage of foreign service is more than compensated by the constant removal of the weak and sickly men by invaliding, both from the army and navy. How largely the apparent rates of mortality are reduced by this process may be inferred from the fact stated by Sir Alexander Tulloch, that among the men discharged from the army on temporary pension, the deaths are no less than 119 per thousand per annum. The amount of loss to the army by death and by invaliding is well illustrated by the series of diagrams by Dr. Farr, which are to be found in the Appendix P. 526.

"*Causes of Mortality*. The causes assigned to us for these high rates of mortality are:—

"1. Night duty.
"2. Want of exercise and suitable employment.
"3. Intemperate and debauched habits among the soldiers.
"4. Crowding and insufficient ventilation, and nuisances arising from latrines and defective sewerage in barracks.

"Without rejecting any cause which may contribute in any degree to the deterioration of the health of the soldier, we are, after a careful investigation of the subject, disposed to attach little comparative importance to the first head. The comparison with the police, who perform a night duty far more severe and yet have a rate of mortality of only one-half that of the infantry of the line, and less than one-half of that of the Guards, seems to support our conclusion. It has been stated, however, that practically the service of the police lasts on an average but five years, owing to the frequency of invaliding or resignation on account of ill health, whereas the average service of the soldier is considerably longer.

"Taking, however, the first five years of a soldier's duty, namely, from 20 to 25 years of age, assuming that some months have been spent in drill, and comparing it with the five average years of the policeman who begins his service at 25 (a comparison in which the relative age is in favour of the soldier), we find that the mortality in the infantry of the line is 17·8 per 1,000, and in the Guards 20·4 per 1,000, against 8·92 per 1,000 in the police.

"It appears, likewise, that when the Guards were engaged in the suppression of the outbreak in Canada, in 1838, their rates of mortality, which at home are in excess of those of the infantry of the line, upon that occasion fell below them. The mortality in the line during that period, in Canada, being 16·5 per 1,000 per annum, and that of the Guards 14·5 per 1,000.

"The second division of the subject assigns a cause of disease and mortality to the existence of which, without the evidence which we are enabled to adduce upon the subject, many would be indisposed to give credence, viz., the want of exercise and suitable employment.

"Of all classes of this country, including trades, occupations, and professions, and including also what may be called the affluent or easy classes, the longest lived is the agricultural labourer. He is neither the best fed, nor the best clothed, nor the best housed man in the country. In many districts he is quoted, and truly quoted, as the reverse. But the one condition in which he differs most from all other classes is the amount and the variety of exercise which he takes in the open air.

"Throughout the tables, showing the rates of mortality in different trades and occupations, those whose avocations require much exercise, whether in doors or out of doors, have almost invariably a decided advantage over those requiring less muscular exertion. The rates of mortality of the cavalry of the line, as has been already shown, are but 13·6 per annum in a thousand, as against 17·9 in the infantry of the line; and this difference, we believe, is in some degree attributable to the greater number of hours passed out of doors on duty by the trooper, the variety of exercise which he takes,—part of his duty being on horseback, part on foot,—and the amount of muscular exertion required of him in grooming his horse, which is far greater than any exacted of the infantry soldier. His stable duty, as well as his sword exercise, calls into activity a different set of muscles to those exercised by men in marching. Indeed, all the attitudes of the foot soldier on parade, at drill, and on the march, are singularly monotonous and constrained, and it is rare to see an infantry soldier whose figure is as fully developed or as well set up as that of the cavalry soldier.

"This is the manner in which his day is spent by the private in the Foot Guards:—

"He gets up at six. There is no drill before breakfast; he makes up his bed and cleans his things; he gets his breakfast at seven. He turns out for drill at half-past seven or eight; his drill may last an hour and a half. If it be guard day there is no drill, except for defaulters. The men for duty are paraded at 10 o'clock; that finishes his day's drill altogether. There is evening parade, which takes half an hour; and then his time is his own till tattoo, which is at nine in winter and ten in summer. That is the day of a soldier not on guard, or not belonging to the company which is out for minié practice.

"Of the hours which the soldier has to himself he spends a very small portion in exercise or out of doors; and Colonel Lindsay, the Quartermaster-General, and Generals Mansell and Lawrence, all recommend that every encouragement and facility should be given to the soldier to practise athletic and out-of-door games and sports, as necessary both for his physical and moral health.

"In the French army these considerations are so entirely recognized, and so great is the importance attached to them, that not only are the soldiers made to pass through a certain course of gymnastic exercises, but among the duties prescribed in the instructions for the medical inspectors is that of inquiring into the practice of these exercises in their districts. We are, therefore, inclined to place want of exercise, and especially of that species of exercise which useful labour supplies, and which would brace and develop the chest and frame, among the causes of the sickness and mortality of the infantry soldier.

"The low rate of mortality in the navy, in which service the men, though necessarily berthed in a very confined space, undergo an immense amount of exercise, calling the greatest variety of muscle into play, and pass a large proportion both of day and night in the open air, appears to favour the opinion we have here expressed.

"As regards the third head, namely, intemperance, if by intemperance be meant drinking to intoxication, we have no reason to think that the soldier is more intemperate than the average of the class from which he originally comes. He has no large sum paid weekly into his hands, with which to spend one or two days in drunkenness, if he were so inclined; nor, with the vigilant discipline exercised over him, could he do so with impunity. But that the soldier frequently indulges in the use of stimulating liquors, to an extent which may injure his health, though not deprive him of his senses, cannot, we think, be denied.

"Mr. Neison states, that the results of an inquiry made into the rate of mortality amongst persons of intemperate habits show that, at ages corresponding to those of soldiers, there is, relatively to the deaths from all other causes, an excessive development of diseases of the "nervous system," and of the "digestive organs."

"In England and Wales these two groups of diseases, at ages from 20 upwards, constitute 15·95 per cent. of the deaths from all causes; but amongst intemperate persons Mr. Neison asserts that they form 50·4 per cent. of all the deaths that take place, being more than three times the general average. These diseases may, according to him, therefore, be regarded, to some extent, as distinctive types of the causes of death amongst intemperate persons, and their paucity or predominance would, in his opinion, fairly lead to inferences as to the general regularity or irregularity of the previous habits of the persons whose deaths they caused.

"But in the service the higher the mortality of the different branches, the greater is the relative amount of deaths caused by diseases of the respiratory organs; in other words, in the household cavalry, which is the healthiest service, the deaths by diseases of the nervous system and digestive organs are as many as one-fifth of those caused by diseases of the respiratory organs, while in the Foot Guards, which is the unhealthiest, they constitute but one-eleventh.

"That the health of the army, like the health of the working classes in this country, would be much improved by greater abstinence from strong liquors, appears to us indisputable. We doubt, however, whether there is now such a difference in the habits of the soldier and those of the labourer or the mechanic in that respect, as would account for the extraordinary excess in the rates of mortality of the former over those of the latter.

"There is, doubtless, a greater amount of dissipation of other kinds among soldiers than among young men of the same class in civil life. Their residence in towns offers great temptation and great facilities for sexual debauchery; and the diseases which are thereby generated,—the existence of which the soldier, from one cause or another, frequently conceals, thereby greatly adding to the intensity of the malady and the difficulty of cure, as well as to the necessary severity of the treatment,—no doubt have a most injurious effect on his constitution.

"There remain to be considered the effects on the health of the army of overcrowding, of insufficient ventilation, of defective sewerage, and of other noxious agencies of analogous character in barracks, and before doing so, we deem it right to call attention to the influence of pulmonary affections on the mortality of the troops, as compared with the mortality occasioned by the same diseases in civil life.

"The following table shows the total annual mortality per 1,000 of the troops of different arms, and of civilians in 24 large towns, at the soldiers' ages, by different diseases, and the proportions of deaths caused in either case, by chest and tubercular, as distinguished from other diseases.

| Ratio of Deaths per 1000 of Mean Strength. | Household Cavalry. | Dragoon Guards and Dragoons. | Foot Guards. | Infantry of the Line. | Civil population of 24 large Towns. |
|---|---|---|---|---|---|
| Inflammation of the lungs, pleurisy, and acute catarrh | ·2 | ·7 | 1·3 | 1·3 | ·5 |
| Spitting blood, consumption, chronic catarrh, asthma, difficulty of breathing | 6·4 | 6·6 | 12·5 | 8·9 | 5·8 |
| All causes - - - - - - | 11·1 | 13·6 | 20·4 | 17·9 | 11·9 |

"From this table it appears that while in civil life at the soldiers' ages, the deaths by pulmonary diseases are 6·3 per 1,000 they amount in the cavalry to 7·3; in the infantry of the line to 10·2; in the guards to 13·8 per 1,000, and that of the entire number of deaths from all causes in the army, diseases of the lungs constitute the following proportion; namely, in the cavalry 53·9 per cent.; in the infantry of the line 57·277 per cent.; in the guards 67·683 per cent.

"It may be stated, that in civil life insufficient clothing, insufficient and unwholesome food, sedentary and unwholesome occupations, and the vitiated and atmosphere of unhealthy dwellings, all contribute to the propagation of this class of diseases. But in the army it cannot be alleged that the clothing, the food, or the nature of the occupation in itself are of a character which would justify the imputation that they are among the predisposing causes of the excessive mortality of the soldier by pulmonary disease.

"One cause alone, viz., a vitiated air, acts with such intensity, especially when superadded to a certain amount of exposure, as not only to produce in the Foot Guards an amount of the disease in question, which is greater than is produced in civil life by all the four causes united, but which actually carries off annually a number of men in the infantry nearly equalling, and in the Guards actually exceeding, the number of civilians of the same age who die of all diseases put together.

"Even here again, it must, however, be remembered that the returns of death occasioned by pulmonary affections in the army do not present the whole amount of mortality caused by this class of diseases, a large number of men being constantly invalided and discharged from the army when incapacitated for duty by the progress of the malady.

"It has been stated to us as a fact so singular that it deserves further inquiry, that the only army in which the mortality does not much, if at all, exceed that of the population from which it is drawn, so far as the latter can be ascertained, is the native army in India, whose rates are slightly lower than those of the native civil population of those districts in which the rates of mortality are known.

"It is also the only army with which we are acquainted which is not barracked. The sepoy receives a small sum under the name of hutting money, with which each man erects for himself a rude construction with mats, and other articles of a similar character; and he frequently sleeps outside of the hut so erected.

"It is also a fact worthy of notice, that the mortality of the army when hutted before Sevastopol in 1856, as compared with that of the troops at home, was nearly one-third less than the mortality of the infantry of the line, and two-fifths less than that of the Foot Guards when barracked in England. The numbers are as follows:—

"The mortality of the army before Sevastopol, during 22 weeks ending May 31, 1856, was, including deaths by violence or accident, at the rate of but 12·5 per 1,000 per annum, as against 17·9 in the infantry and 20·4 in the Guards when quartered in England.

"Perhaps no army was ever better cared for, or more sanitary precautions taken in its behalf as regards drainage, both of surface and subsoil, cleanliness, ventilation of huts, diet, clothing, etc., than the army before Sevastopol, during the period mentioned. In the month of May alone the mortality was at the rate of only 8 per 1,000 per annum.

"Again, the cavalry at home, whose rates of mortality are lower than those of any of Your Majesty's troops, are generally less crowded in their barracks than the infantry; the reduced strength of the cavalry regiments leaving greater space for the accommodation of each man.

"In considering the effect on health of the constant breathing of a vitiated atmosphere in barracks, it must be borne in mind, that 57·277 per cent. of the deaths in the infantry of the line are caused by diseases of the respiratory organs, and of the Guards no less than 67·683 per cent.

"*Barracks.* The dormitories or barrack rooms are very confined, the minimum cubic space allowed to each soldier by regulation being only 450 feet, and in a majority of cases even this minimum is not attained, and in a number of barracks there is a deficiency of one-third, and in some instances of more than one-half of the space allotted by regulation. Sir John M'Neill states, that the pauper in the Scotch workhouses is allowed 480 feet per bed, and not only is this minimum rigidly insisted upon, but the houses being scarcely ever full it is practically much exceeded, and the pauper is never in his dormitory during the day. In the barrack, on the other hand, it is stated by some non-commissioned officers examined by the Barrack Committee, that even the regulated space of one foot between the beds is practically unattained; and all experience goes to prove, that the amount of interval allowed between the beds is of more consequence, so far as health is concerned, than the mere amount of cubic space given above by the altitude of the room. Owing to the ordinary construction of the buildings, the barrack rooms very seldom have windows at opposite sides or ends of the room, and there are, consequently, very insufficient means of ventilation, though the number of men sleeping together in a confined space renders every access of fresh air the more necessary. Even where ventilators exist, they are frequently stopped up by the men themselves, who come from a class very little persuaded of the advantages of ventilation, and whom poverty has accustomed from their youth up to look to the exclusion of the external air, in the absence of fuel, as the best mode of securing warmth. Barrack-rooms are occasionally found in the basement of the building, approached by descending steps from the natural surface level, the tops of the windows, which open on one side only of the rooms, being little, if at all, above such surface level; and in low rooms thus situated a number of men may be found lodged in beds so closely ranged that the side of one touches the side of the other. The result is, that the soldier sleeps in a fœtid and unwholesome atmosphere, the habitual breathing of which, though producing for the most part no direct immediate effects, probably lays the seeds of that pulmonary disease which is so fatal in the British army. Of the state of this atmosphere, abundant evidence, though of a most disgusting nature, is to be found in the statements of the witnesses before the Barrack Committee.

"Colour-serjeant Reynolds being asked, 'Did you observe any ill effect arise to the health of the men, or to their spirits, in consequence of the atmosphere in which they were shut up at night?' says, 'There used to be frequent complaints; there was a good deal of coughing, and it used to cause a phthisicky sensation in the throat, and spitting in the morning.'

"It must be remembered that in the barrack-rooms, the atmosphere of which we have described, the men are expected to take all their meals, and the number of hours they spend in them during the day makes their thorough purification before the return of night almost impossible.

"*Encampments.* Under the existing system the medical officer is required by the regulations to examine the ventilation and cleanliness of the barracks and quarters, and to suggest improvements to the commanding-officer, sending a copy of such suggestions to the Director-General. He is also required, upon the occurrence of cholera or any other formidable disease, to report to the Director-General his opinion as to its cause, and the treatment adopted. But no power is given him to carry into operation any of the measures he may deem necessary, nor is there any regulation authorising him to press upon his commanding officer the adoption or even the consideration of his suggestions, if the latter be not disposed to consult him. There is in this respect a total, and, as it appears to us, injurious want of connection betwen the medical and executive departments of the army.

"*Ration.* The ration of the British soldier consists at home of 1 lb. of bread and $\frac{3}{4}$ lb. of meat, at which rate it was fixed so far back as 1813, and has never since varied, except that by a warrant dated February 1833, an additional $\frac{1}{2}$ lb. of bread was given to troops encamped in England.

"Abroad, the ration consists of 1 lb. of bread or $\frac{3}{4}$ lb. of biscuit, and 1 lb. of meat, either fresh or salt, the additional $\frac{1}{4}$ of a pound being given to compensate for the inferior quality of foreign compared with English meat. There are, however, two exceptional rations still given, one at Hong Kong, and the other at certain out-post stations at the Cape of Good Hope, where additional articles are supplied, for which the soldier pays one penny at the former, and three half-pence at the latter station.

"But the $\frac{3}{4}$ lb. of meat and 1 lb. of bread at home, and the 1 lb. of meat and 1 lb. of bread abroad, with the stoppages of $4\frac{1}{2}d.$ in the one case and $3\frac{1}{2}d.$ in the other, by no means represent either the amount of food daily consumed by the soldier, or the amount of money which he pays for it. Indeed a mere bread and meat ration, even if increased in quantity, could never ensure health, without the addition of vegetable food.

"Some years ago the practice of having a third meal in the afternoon was introduced into some regiments by commanding officers, who paid much attention to the health and well being of their men, the expense being defrayed by a stoppage made by consent in the regiment. Up to that time the soldier had but two meals, breakfast at half-past 7, and dinner at half-past 12; there remained therefore an interval of nineteen hours during which the soldier might be altogether without sustenance; and though in most cases he probably obtained a meal at his own expense, the soldier had no guarantee that he got it at the lowest cost, nor had the public any security that the meal was the best or the wholesomest that could be provided for him.

"The good effect of the introduction of this third meal on the discipline as well as the health of the men was remarkable. The meal acted as a roll-call, bringing men back into barrack towards evening; while the substitution of solid food for the dram to which exhausted men had recourse diminished drunkenness.

"*Cooking.* At present, in barrack as in hospital, but one mode of dressing food is recognised or provided. Coppers for boiling exist in every barrack, but no meat can be baked, roasted, stewed, or fried.

"When a soldier, therefore, enters the service, he has the prospect of dining on boiled meat every day for twenty-one years, if he is enabled to serve so long. There can be no doubt that some variety, not only in the material, but in the preparation of food, is necessary to ensure its being palatable; and we have it stated in evidence that men frequently leave part of their meat, which, when cooked and free from bone, does not much exceed half a pound, their stomach loathing the constant repetition of the same food in the same form.

"The experience of Dr. Balfour at Chelsea shows that a variety in the mode of cooking the food consumed has produced a higher standard of health among the boys in the Royal Military Asylum, with a positive diminution in expense. That the men themselves attach much importance to obtaining this variety is proved by the fact that they send out their meat on certain days in the week, at their own expense, to bakers in the towns where they are quartered, in order to have it cooked differently from what can be done in the barrack kitchens.

"*Clothing.* Of late years great improvement has been made in the clothing of the troops. The form of tunic now adopted affords protection to the hips and belly of the wearer which the coatee did not. The material is stated to be better than that formerly in use, and the fashion of tight clothing is for the present at least discarded. Too much importance cannot be attached to an easy adjustment of the clothing, so as to leave to the respiratory and other organs of the body, as well as to its muscular development, the utmost freedom. Great benefit is stated to have resulted from the use of the canvas frock at the Cape and in the Crimea, in lieu of the cloth tunic. As regards under-clothing, the balance of evidence, both of commanding and medical officers, is in favour of the flannel shirt. But the opinion of the non-commissioned officers examined was decidedly unfavourable to it, principally on the ground of the increased cost of the article, which is one of the necessaries kept up at the expense of the soldier.

"The quality of the boots is complained of, and it has been suggested that a boot should be adopted, capable of being laced over the trouser, as the French gaiter is buttoned on the march, so as to prevent the accumulation of wet and mud on the loose trowser round the ankle.

"The stiff stock is condemned by almost every one who has given his opinion upon it.

"But the most difficult problem to solve in army costume is the head-dress. Formerly it was a *sine quâ non* that the head-dress should protect the head of the wearer from a sabre-cut. It was looked upon as armour still used for the protection of the head. We doubt the value of this protection, if it be purchased at the expense of a degree of weight and of heat likely to prove injurious to far more men than it will ever preserve from sabre wounds. The altered system of warfare, from the daily increasing range of the weapons used, renders the attempted protection of particular parts of the body more than ever useless ; aad it becomes the more important to consider what character of head-gear will afford the best protection to the head, not from sabre or bullet, but from heat, cold, and wet, and can be worn most conveniently by the soldier on the march or when sleeping at the bivouac.

"Much complaint has been made by witnesses of the form of the peak of the forage-cap (now no longer worn), but which had none of the advantages of a peak, as it either afforded no protection to the eyes, or, by coming down close upon them, created so heated an atmosphere as sensibly to incommode them.

"This applies, though in a less degree, to the shako now in use.

"The busby is stated, from its form or want of form, to press in a narrow circle on the man's forehead to a degree very painful to the wearer. There is no reason why the interior of the busby should not be adapted to the form of the human head, though the exterior should still preserve its rectilinear shape.

"We are satisfied that the shako is, from its colour, weight, material, and form, altogether inapplicable to tropical climates such as those in which some of our troops psss a great portion of their lives. For them there is no head-dress giving so efficient a protection to the head, face, and neck as a light cap covered by wadded linen, with a flap hanging down behind, or else a few yards of linen rolled, turban fashion, round the forage cap; and we recommend the adoption of some such head-dress in India and all climates of similar temperature.

"The great coat worn in the British army is of very bad material, of little use against cold, while it readily imbibes and retains wet.

"*Effect of Sanitary Measures.* It ought not to be possible to say, as was said by a witness whom we examined, that a soldier never knows a healthy home, as regards air and space, till he commits some crime which brings him into the thoroughly ventilated cell of a military prison; or, as another witness stated,—

"'That a soldier's barrack room at present has not the least pretension to the comforts of an ordinary dwelling-house, and, what is infinitely more disgraceful, there is not even the attempt made to introduce into it the decencies of civilized life.'

"The strongest example of what well-directed care and precaution of a sanitary kind can effect, is to be found in the comparative health, at different periods, of the army in the Crimea. The example is of singular virtue, inasmuch as it offers to our view the most complete case on record, on the largest scale, of neglects committed, of consequences incurred, of remedies applied, and of consequent improvements in health and efficiency. Without entering into any of the controversies now we trust at an end, as to what was or what was not avoidable in a winter campaign, with an army unacclimated to war during a siege unexampled in its character, the facts remain, and it is our duty not to neglect the lessons that may be drawn from them.

"Throughout the winter of 1854-5 the troops were suffering from work altogether disproportioned to their strength, from broken rest, insufficient clothing and shelter, unwholesome food, and want of cleanliness. As the spring advanced, to these causes of disease and mortality were added others, arising from want of drainage and ventilation, and the nuisances resulting from the lengthened occupation of the same ground, without sufficient countervailing precautions. Throughout the period of seven months, from October 1, 1854, to April 30, 1855, the rate of mortality rose as high as 600 per 1,000 per annum. But in November and December of 1855, with supplies abundant, food of a wholesome character, and improving sanitary conditions, the rate of mortality per 1,000 per annum had already fallen to 44 and 33; and with huts well drained and ventilated, nuisances removed, and the camps thoroughly cleansed, from January to May, 1856, the rate of mortality of the army in the Crimea per 1,000 per annum fell to 12½ and in May to 8, as against 17·8 in the infantry at home. Diagrams H. I. and K., Appendix No. lxxii., illustrate the variations in the rates of mortality in the army in the East during the Russian war.

"*Hospitals.* Before discussing the changes which we think requisite in the organization of army hospitals, it is desirable to give a short account of the existing hospitals, and the system on which they are managed.

"The army general hospitals in Great Britain and Ireland are three in number, and are situated at Chatham, Dublin, and Cork; but the latter is on a most limited scale. There are none in the colonies nor in India, except the Company's general hospital existing at each of the three presidencies, and such temporary general hospitals as may be formed with armies in the field.

"Besides these, there are others equalling the general hospitals in extent, such as the hospitals at Dublin, Woolwich, Portsmouth, Plymouth, Aldershot, and Shorncliff; these, however, are not general hospitals, but merely congeries of several regimental hospitals, each remaining as completely distinct, in point of medical and other attendance and supervision, as though placed in the separate barracks in which their respective regiments are quartered.

"*Military Hospitals at Home.* The contracted space and want of ventilation in our military hospitals do not give to the patients the same chance of recovery as is afforded by the better accommodation of the naval and civil hospitals of this country.

"*Field Hospitals.* We would here observe, that in no case when it can possibly be avoided should the sick be treated in bell tents. They are described as hot in summer, wet in autumn, cold in winter, unpleasant during windy weather, too confined for the performance of professional duties, ill adapted to the nursing of sick men, and far too limited on the floor to enable medical officers to render patients in any degree comfortable. On the line of march they may suffice as a shelter for transient and slight cases.

"On the other hand, marquees can be made comfortable in a standing camp by means of portable bedsteads arranged on each side, with a passage along the middle. They are temperate in hot weather, dry when it rains, moderately comfortable during the prevalence of high winds, afford greater shelter than bell tents against cold, enable medical officers to approach the patients with more comfort to themselves, and permit the orderlies to attend better to the wants of the sick. But it is of the utmost importance that they should be thoroughly ventilated, in which respect there is great room for improvement.

"*Returns, Registers, &c.* Our attention has been drawn to the complaints, made by the medical officers, of the great amount of clerical labour thrown upon them by the cumbrous nature of the returns required of them, and the unnecessary writing caused by the unceasing and frequent recourse to the system of requisitions.

"It appeared to us that the true way of diminishing the necessity of a frequent recourse to requisitions upon the purveyor is to introduce variety in the ordinary hospital diets, on which subject we have already made a recommendation, so as to comprise under the head of a diet such of the extras as are now the most usual subjects of requisition; indeed, it not unfrequently happens that a patient is continuously and almost entirely dieted on extras, which must, nevertheless, be the subject of daily requisition. As the diet table is as complete a voucher to the purveyor, this change would greatly diminish the amount of writing and the number of separate transactions involved by a recourse to requisition, without in any degree diminishing the financial check upon the expenditure, which we consider it a paramount necessity to maintain.

"*Statistics.* The ultimate fate of an army frequently depends more on the intensity of its diseases and upon their causes than on their extent; and it is important that the Commander of the Forces should know what proportion of his disabled force is suffering from wounds and what from disease, and of the latter what proportion is attributable to that class of maladies which experience has shown can be greatly diminished in severity by sanitary measures. For this purpose an improved classification of diseases should be adopted in the regimental 'weekly states,' whereby the zymotic diseases would be shown in a group by themselves; and every medical officer should be required, as is now sometimes done, to write upon the "state" the sanitary or other defects which may have predisposed the men to such diseases.

"Dr. Farr, who is the head of the Statistical Department of the Registrar-General's office, informed us that the Registrar-General is anxious to incorporate with his periodical reports the mortality of the British army, so as to include in one view the deaths of all classes of Englishmen, whether at home or abroad, who have not, like emigrants or exiles, severed the tie which bound them to their own country.

### SUMMARY OF RECOMMENDATIONS.

"*Barracks, &c.* We recommend that in future a minimum cubic space of not less than 600 feet be allotted to each man in his barrack-room and in the guard-room, and that an interval of at least three feet be allowed between each bed in the former.

"That urine-tubs be on no account tolerated in the barrack-rooms; but that urinals with a water supply outside the barrack-room, or common chamber utensils, be substituted.

"That every barrack should contain ablution rooms and baths, laundry and drying-room, and workshops; and also, that suitable provision be made for non-commissioned officers' and married soldiers' quarters.

"That day-rooms be constructed for the use of the men in some of the principal barracks at home, and, if found advantageous, that they be extended to all barracks.

"That all barrack-rooms, guard-rooms, and day-rooms be sufficiently warmed and lighted, whatever may be the number of men occupying them; and that gas be used for lighting whenever it is obtainable.

"That, with a view to enable the soldier to have variety in his meals, the means of baking, frying, &c., be provided in the kitchens of all barracks and hospitals.

"That a Commission be immediately appointed, consisting of an engineer officer, and a medical officer versed in the practical application of sanitary science, who, together with the medical officer and the barrack-master of the inspected barrack, shall undertake the inspection of every barrack, and execute the necessary works, until every barrack in use shall, in its turn, have been brought to a healthy and satisfactory condition; and that in the meanwhile, immediate orders be given to the barrack department to revise the allotment of men to rooms, so as to divide the cubic space more equally.

"That, in future, before a barrack is built, the advice and opinion of competent medical officers be taken as regards both the site and plan.

"That, in order to secure that sanitary considerations shall not be overlooked in the choice of sites for encampments, hospitals, barracks, or in any matter involving the health of the troops, such as water supply, drainage, food, clothing, &c., medical officers be invariably consulted; and in order to fix on commanding officers and on medical officers the responsibility properly belonging to each, that the medical officer shall be required to give his advice in writing, the commanding officer to affix in writing his reasons for rejecting it, if he think fit to do so, and to transmit the document to superior authority.

"That, in order to secure to the commanding officer of an army in the field the most efficient sanitary advice, and to relieve the principal medical officer of duties which his other avocations leave him no time to perform, a sanitary officer be appointed to act under the authority of the principal medical officer, but to be attached to the staff of the quartermaster-general.

"That facilities and encouragement be given for all athletic games, such as fives, cricket, quoit, single-stick, for gymnastic exercises, &c., and that the men be employed on different kinds of labour when possible.

"*Rations and Clothing*. We recommend the consolidation of the present stoppages for ration and regimental messing, and, in consideration of that consolidated stoppage, the issue to the soldier by the commissariat of a sufficient amount of food for three daily meals, including vegetables, tea, coffee, and sugar, with such variations abroad as the climate or the market may render necessary.

"That the soldiers' 'necessaries,' hitherto obtained by regimental contract, be provided by the commissariat.

"We recommend the adoption of a better boot; also a lighter stock of the pattern specified; a more horizontal peak to the forage cap; and the substitution of a light cap, with a wadded linen cover or a roll of linen, for the shako in hot climates.

"That greater attention be paid to the interior form of the head-dress, whether shako or busby; that the great coat be of a better quality and more durable texture; that all clothing be made sufficiently loose to permit the free use of the limbs and unimpeded action of the muscles, and that the material of the clothing be varied according to the climates in which the troops may be serving.

"We strongly recommend the immediate testing, by its issue to half a battalion, of the 'Berrington' knapsack sling.

"*Hospitals*. We recommend that in all cases competent medical sanitary officers should be consulted as regards the selection of site, and the original construction of hospitals to be built.

"That the present minimum space allowed by the regulations to each bed be increased from 600 feet at home and 800 in tropical climates to 1,200 at home and 1,500 in tropical climates; and that a minimum distance of 4 feet between the sides and 12 feet from foot to foot, and as much more as the size of the ward will allow, be maintained between the beds.

"In the construction of new hospitals, we recommend the plan of separate pavilions, with lateral windows on opposite sides, and natural ventilation.

"We recommend the use of Parian cement or other impervious material for the walls and ceilings of hospital wards, in lieu of bare brick or plaster.

"That sufficient provision be made for warming and lighting the hospital according to the weather and season, on the requisition of the medical officer in charge of the ward.

"That water-closets be built in connexion with every hospital, and an efficient sewerage effected by impervious drains not passing under the building, all cesspools in the immediate vicinity of hospitals being done away with, and that the building containing the closets and sinks be cut off from the hospital by a ventilated lobby.

"That suitable lavatories, baths, and laundries be provided.

"That the staircases and landings be in all cases constructed of stone.

"That proper ranges and ovens be put up in the kitchens of every hospital, so that meat may be stewed and roasted as well as boiled, and a sufficient variety in the preparation of food may be afforded to the sick.

"*Returns, Registers, Tables.* We recommend the adoption of a form of monthly diet table, by which the necessity of re-writing every day a nominal list of every patient in the hospital will be done away with. And we recommend that all the regulations affecting the army medical service be revised, many having become inapplicable owing to the changes in the organisation of the War Department, and from other causes. The sanitary directions which they contain are neither numerous nor specific enough, and presuppose a much larger amount of practical sanitary knowledge than is usually possessed by most medical men, either in or out of the army. There are also no means provided whereby any recommendations, however good and appropriate, for securing the health of the troops can be brought into practical application.

"*Statistics.* We recommend that a nominal list of the deceased soldiers, and of births and marriages in the army, be communicated to the Registrar-General at such periods and in such a shape as may be necessary for the objects we have indicated.

"That an improved nomenclature of diseases be adopted in the Army Medical Returns, and such alteration in the classification of diseases as may admit of an accurate comparison of them with other returns of a similar nature; and that provision be made for the periodical publication of the statistics of sickness and mortality among the troops.

"That forms be prepared for the use of the medical officers, which shall enable the commander of the forces in the field to know what is the past and existing rate of sickness and mortality in his army, what are the causes of it, and for what time, at a continuance of the same rate, and with what reinforcements he can maintain his army in the field. These forms should besides enable the home authorities to compare the rates of the army with those of civil life and with its own rates at previous periods.

"That the household brigade be directed to furnish to the Army Medical Board the same returns of sickness and mortality as the rest of your majesty's troops.

"For the attainment of these objects we recommend the formation of a statistical branch in the Army Medical Department."

In the way of comment on this report, but little remains to be said. The whole is characterised by a broad and liberal feeling, and an earnest desire for the progress of that greatest of all the practical sciences, over which the goddess ῾ΥΓΙΕΙΑ mythologically reigns. There is common sense reiterated in every paragraph, till the grand faculty strikes like absurdity. But while there is little to challenge, the Commissioners, we think, are grievously wrong in one particular, viz., in endeavouring to define too sharply the differences between preventive and curative medicine, *i. e.* in looking on hygienic medicine as a specialty. Into this error they have been unfortunately led by the evidence of one or two of the witnesses who appeared before them, to whose obscurity hygienic principles, as part and parcel of a reformed system of medical education and medical practice, have not yet appeared, and, we fear, never will at this late hour appear in the light of demonstration.

The error to which we here refer is given in page xxi of the report. We quote the passage in its entirety.

"In civil life sanitary science as yet is neither much studied nor widely spread, nor has the value of its practical application to the ordinary conditions of life obtained any very general acquiescence. While the tendency to fuse together the practice of medicine and surgery has thrown almost the whole practice of the country (except that of the great towns) into the hands of the general practitioner, a subdivision of labour of another kind has simultaneously been gaining ground in the medical profession. The study of sanitary science has been taken up as a specialty, and the field has been abandoned by the mass of the profession to be exclusively occupied by those who so study it. The names of those eminent in either branch are perfectly well known to the public, who employ the one or the other according as they want individual sickness treated or public sickness prevented. It is rare to send for the health officer to treat sickness, or to employ the eminent practising physician or surgeon to drain a town, or to guard a district against the approach of cholera.

"The fusion between the medical and surgical specialities is, in the Army Medical Department, even more complete than in the civil profession; and if efficient sanitary officers are to be obtained, it will be by the encouragement offered by Government to the army medical officers to make themselves thoroughly masters of the specialties of that branch of the medical art and its practical application."

As advocates of the unity of sanitary and curative medicine, we join issue with every sentiment in this paragraph as at once unreasonable and mischievous. We confess that, by mere accident in the progress of medicine, a few estimable Esculapians did, some time ago, isolate themselves from their brethren to become the founders and forerunners of the hygienic reform in physic; but the mission of these men, though effective, has been short. They have not increased as specialists, because their arguments have convinced the profession that the principles they advocated are most exemplary, true in every particular but one, in that, viz. of considering sanitary studies apart from the study of disease at the bedside; eventually, as a class, they will be lost altogether in the universal body which shall accept both curative and preventive medicine in their unity.

This very report, indeed, proclaims the unity we describe. It tells us an all-important fact about the prevalence of pulmonary consumption amongst soldiers, and tells, too, the cause of that prevalence. Is it that this fact shows only how an army may be saved from that disease? is it not, also, that it indicates how a nation may be cured of the disease? For our own parts, and in the name of the largest constituency in this country of sanitary students, we protest against the division of medicine, and for the unity of our science in all its branches. We maintain, on the one hand, that a man ignorant of the phenomena of disease can know not so much as the elements of sanitary science. We affirm, that an immense amount of mischief has been perpetrated by amateur attempts at sanitary pursuits. We assert, on the other hand, without any fear of noticeable contradiction, that whoever assumes to meet disease, and to fight the practical fight with death, without being well grounded in a knowledge of the grand phenomena of life and the general causes of disease,—in other words, without information on the basic points of the sanitary laws,—is one who can only trifle with secondaries, who can see no rational way to treatment, who can but move on, trusting to the ignorance of the world, imposing on himself and imposing on all who may blindly intrust their lives to his unskilful and boastful rashness.

For the rest, we believe the authors of the report are unanswerable in their arguments, and the country owes them a debt not readily repaid for the care, manliness, and industry which have characterised their eventful inquiries.

